# Measurement of Quality of Life III. From the IQOL Theory to the Global, Generic SEQOL Questionnaire

**DOI:** 10.1100/tsw.2003.77

**Published:** 2003-10-13

**Authors:** Soren Ventegodt, Joav Merrick, Niels Jorgen Andersen

**Affiliations:** The Quality of Life Research Center, Copenhagen K, Denmark

**Keywords:** Quality of Life, QOL, SEQOL, QOL5, measurement, human development, holistic medicine, public health, Denmark

## Abstract

The Danish Quality of Life Survey is based on the philosophy of life known as the integrative quality-of-life (IQOL) theory. It consists of eight different quality-of-life concepts, ranging from the superficially subjective via the deeply existential to the superficially objective (well being, satisfaction with life, happiness, meaning in life, biological order, realizing life potential, fulfillment of needs, and objective factors [ability of functioning and fulfilling societal norms]).This paper presents the work underlying the formulation of the theories of a good life and how these theories came to be expressed in a comprehensive, multidimensional, generic questionnaire for the evaluation of the global quality of life — SEQOL (self-evaluation of quality of life) — presented in full length in this paper. The instruments and theories on which the Quality of Life Survey was based are constantly being updated. It is an on-going process due to aspects such as human development, language, and culture. We arrived at eight rating scales for the quality of life that, guided by the IQOL theory, were combined into a global and generic quality-of-life rating scale. This was simplified to the validated QOL5 with only five questions, made for use in clinical databases. Unfortunately, the depth of human existence is to some extent lost in QOL5.We continue to aim towards greater simplicity, precision, and depth in the questions in order to explore the depths of human existence. We have not yet found a final form that enables us to fully rate the quality of life in practice. We hope that the several hundred questions we found necessary to adequately implement the theories of the Quality of Life Survey can be replaced by far fewer; ideally, only eight questions representing the eight component theories. These eight ideal questions have not yet been evaluated, and therefore they should not form the basis of a survey. However, the perspective is clear. If eight simple questions can accurately rate the quality of life as well as its depth, we have found an instrument of immense practical scope.

### INTRODUCTION

The Danish Quality of Life Survey is based on the philosophy of life known as the integrative quality-of-life (IQOL) theory. It comprises eight different quality-of-life concepts, ranging from the superficially subjective via the deeply existential to the superficially objective.

This paper presents the work underlying the formulation of the theories of a good life and how these theories came to be expressed in a questionnaire for the evaluation of the quality of life, SEQOL. We arrived at eight rating scales for the quality of life that, through the analysis of the responses, were combined into a global and generic quality-of-life rating scale.

This paper thus presents an important aspect of our work with the quality-of-life (QOL) concept through the last decade. We have developed the quality-of-life philosophy[1,2]; the SEQOL, QOL5, and QOL1 questionnaires[3,4,5]; the quality-of-life theory[4,5,6], the quality-of-life research methodology[6,7]; and we have carried out quality-of-life population surveys[8,9,10,11,12,13] and developed techniques for improving quality of life with chronically sick patients[14,15]. A comprehensive presentation of our research approach can be found in our paper[16].

### FROM QUALITATIVE TO QUANTITATIVE QUALITY-OF-LIFE RESEARCH

Before reaching the actual formulation of the eight quality-of-life theories on which the questions of the questionnaire were based, we spent a long time conducting qualitative research on the quality of life. As mentioned in the introduction, such a dialogue between qualitative and quantitative methods is crucial for quality-of-life research.

A long period of informal and casual discussion with various groups of selected people preceded the Quality of Life Survey. This discussion turned into interviews on the quality and content of life and meaning in life with fixed sequential questions that seemed crucial and relevant. These questions gradually led to a formal questionnaire, which has been amended again and again as more interviews and discussion with critical respondents, including colleagues and lay people, have been carried out during the developmental phase.

The questionnaire we now use has been through 20 pilot versions over a 3-year period, and was handed out to many different groups of people during that time. The reason for so many pilot versions was that we wanted to create it from scratch, including new, improved ratio scales, using a new methodological concept[6,16], to be absolutely certain that the global quality-of-life data we extracted from 10,000 Danes were scientifically valid, and also sufficient for testing in a prospective study. The research hypothesis was that poor global quality of life is the curse of many modern diseases.

Among the groups were 20 plastic surgery nurses, 40 university extramural quality-of-life course participants, 120 patients in the Department of Dermatology at the University Hospital in Copenhagen, 15 members of a study group on quality-of-life research based in Copenhagen, 50 students at *folkehøjskole* (institutions of popular education), and 200 people randomly selected from the Civil Registration System (CPR Register) in Denmark.

During this period, the wording of the questionnaire was refined and the eight basic theories and their organization into an integrative theory were subjected to constant re-evaluation[4,6].

The results were hard earned and express some of the theoretical adjustments that invariably take place when a major project is launched. Reality is always far more disordered and complex than anticipated, and we experienced this during this project. This meant that, in relation to the theoretical side of the Quality of Life Survey, the eight theories could not be fully in place for the initial formulation of the questions. The scale was adjusted as it was used, providing us with feedback on the value of the theories on which they were based, such that the theories too were constantly being developed. It was a complex task to convert the eight theories from the integrative theory into a quality-of-life scale[6].

### EIGHT QUESTIONS BASED ON EIGHT THEORIES

Definitions of what makes up a good life are bound to be subject to numerous and diverging interpretations. The IQOL theory, explained in detail in another publication[17], states that the quality of life emanates from[4,5,6]:

1. Well being[6,17]
2. Satisfaction with life[6,17]
3. Happiness[6,17]
4. Meaning in life [5]
5. Biological order[6]
6. Realizing life potential[18]
7. Fulfillment of needs[19]
8. Objective factors (ability, societal norms)[6,17]

The theories, described in detail elsewhere [5,6,17,18,19], led to the development of SEQOL (see Appendix) and later the short version, QOL5[4]. The short version was very much needed for use in clinical databases, where the resources are too limited for using a comprehensive questionnaire like SEQOL. Other short questionnaires like Nottingham Health Profile and Sickness Impact Profile are generic in that they can be filled in by everybody no matter which gender, culture, or age, as shown in another study[3], but not global, i.e., not expressing the total and overall quality-of-life state of the person. The QOL5 questionnaire is very practical, but unfortunately leaves out much of the existential depth of a human being. The question is then: is it possible to construct a simple, common method to assess or rate the quality of life as understood using the eight theories, with full respect for life and its complexity?

In developing and applying the rating scale, the reduced combination scale[20], the first task was to formulate an individual question with the purpose of making the respondents rate their own quality of life. The simplest versions of the eight questions, one derived from each of the eight theories, are listed below:

Q. 1 How are you **feeling** now?

1 Very good
2 Good
3 Neither good nor poor
4 Poor
5 Very poor

Q. 2 How **satisfied** are you with life now?

1 Very satisfied
2 Satisfied
3 Neither satisfied nor dissatisfied
4 Dissatisfied
5 Very dissatisfied

Q. 3 How **happy** are you now?

1 Very happy
2 Happy
3 Neither happy nor unhappy
4 Unhappy
5 Very unhappy

Q. 4 How **meaningful** is your life now?

1 Very meaningful
2 Meaningful
3 Neither meaningful nor meaningless
4 Meaningless
5 Very meaningless

Q. 5 How **balanced** (your inner equilibrium and state of health) are you now?

1 Very balanced
2 Balanced
3 Neither balanced nor unbalanced
4 Unbalanced
5 Very unbalanced

Q. 6 How well are you realizing your **deepest dreams and desires** now?

1 Very well
2 Well
3 Neither well nor poorly
4 Poorly
5 Very poorly

Q. 7 How well are your **needs** being fulfilled now?

1 Very well
2 Well
3 Neither well nor poorly
4 Poorly
5 Very poorly

Q. 8 How many of the following **societal norms** do you fulfill now? (Societal norms here means having [a] a job; [b] education; [c] nuclear family; and [d] at least two friends in whom you can confide everything and do so.)

1 All four
2 Three of four
3 Two of four
4 One of four
5 None

Most of these questions focus on the central theme of this theory: well being becomes "How are you feeling?", fulfillment of needs becomes "How well are your needs being fulfilled?", and so forth. We add "now" because we want a current evaluation, not an evaluation of one's entire life up until now. Current evaluation through repeated surveys of the same respondents enables us to study changes in the quality of life and compare these with other changes in life, which again may indicate dynamic relations between the quality of life and other aspects of life.

### ARE THE QUESTIONS SCIENTIFICALLY ACCEPTABLE ?

Do these questions operationalize the eight theories properly? Are they satisfactory rating scales for the quality of life?

#### Theory 1: Well Being — *"How are you feeling now?"*

With the response options ranging from "very good" to "very poor", scored in conformity with the reduced combination scale outlined in Ventegodt et al.[20], the question is a straightforward and excellent application of the theory.

#### Theory 2: Satisfaction with Life — *"How satisfied are you with life now?"*

This is also a simple and straightforward application of the theory.

#### Theory 3: Happiness — *"How happy are you now?"*

"Happiness" is seldom used in everyday language, but it is well known, idiomatic, easily understood, and produces a satisfactory question.

#### Theory 4: Meaning of Life — *"How meaningful is your life now?"*

Even if "meaning in life" can be understood, not many people in our culture have thought much about it. It is therefore not well suited to a broad population survey. Furthermore, this is a very private sphere, where people might be easily embarrassed. The question on meaning in life touches on religious themes, and in a relatively nonreligious country like Denmark it easily causes superficial, routine responses or attempts to justify oneself, as when people are asked why they go to church on Christmas Eve and not during the remainder of the year. Another problem is the risk of annoying the respondents by questioning them on such nonrational matters in a scientific survey.

Indeed, the researcher quickly finds him- or herself in a methodological minefield in which the quality of the responses may be compromised. We were aware of this prior to inaugurating our Quality of Life Survey. The question was thus not asked directly, but as part of a long series of questions that indirectly served to rate the quality of life. Further, the analysis showed that the series of questions did not act quite as expected statistically, which meant that we faced difficulty.

In a subsequent analysis, we chose to omit this theory from the rating scales chosen and used a substitute instead. We will elaborate on this below. The question asked on how meaningful one's life is does not function very well linguistically as regards to the two final response options "meaningless" and "very meaningless". These two alternatives are identical in meaning. However, the symmetrical scale in conjunction with the modifier "very" solves this problem in practice, so that it becomes clear to the respondent what is meant by the expressions[6]. The concept of meaning in life might very well become a common phrase and thus part of our daily world. It is therefore possible that it may be included in a questionnaire survey in Denmark in the near future. The quality-of-life concept has gone through this process within the past 5–10 years.

### Theory 5: Inner Balance and Biological Order — *"How balanced (your inner equilibrium and state of health) are you now?"*

The theory on the biological information system presupposes expertise and thus needs to be expressed in more general terms here. We have therefore clarified the question by supplementing it with an explanatory statement in brackets ("inner equilibrium and state of health"), and we hope that this makes the theory accessible to the layperson. Still, the question was not wholly satisfactory and was therefore not included in our Quality of Life Survey. Instead, we asked our respondents to evaluate their present mental and physical state of health.

In earlier pilot projects, we had confidence in the value of a series of questions on illness and health problems, but concluded that these questions merely indicated the state of affairs of the biological information system. The series of questions did not generate an evaluation of the biological order; they only indicated its existence. Three facts also served to indicate the disadvantages of using illnesses and health problems as a measure of biological order: first, the scale used was not a ratio scale; second, not only physical but also mental and social aspects of health and well being play an important part of the biological order in the broad sense as we use it; and third, it might be a good idea to omit this assessment of state of health from the quality-of-life rating scales and have it be an independent factor whose link with the quality of life can be investigated.

We finally concluded that the theory of biological order cannot be used satisfactorily, and it was therefore omitted from the quality-of-life rating scales. We explain below how we tackled the problem: we eliminated rating of the deepest layers of the individual.

### Theory 6: Realizing Life Potential — *"How well are you realizing your deepest dreams and desires now?"*

The theory on the quality of life as realizing life potential is closely connected with this research project[1,2,5,6]. Although realizing life potential can be understood, its meaning, as used in this context, cannot be presupposed. It therefore needs to be converted into everyday language.

Asking respondents how well they realize their deepest dreams and desires is one way of finding out to what degree respondents use their life potential to the utmost. We might choose to word the question differently. We chose to divide life into a series of ever more comprehensive domains (self, partners, parents and children, friends and acquaintances, nature and society) and systematically examined the quantitative and qualitative aspects of each of these domains. We are currently investigating whether this question actually works in practice.

### Theory 7: Fulfillment of Needs — *"How well are your needs being fulfilled now?"*

Fulfillment of needs is part of everyday language and is readily understood by most people. However, not everyone agrees on what the needs are. So it is a good idea to ask about certain needs: the need for food, security, etc. based on the individual's personal theory.

### Theory 8: Objective Factors (Ability of Functioning and Fulfilling Societal Norms) — *"How many of the following societal norms do you fulfill now?"*

Just asking people how well they function makes it easy for them to neglect a difficult disagreement with the culture they live in. An important philosophical question is if a healthy person actually would want to be a part of our society. We take the position that a healthy person will fit in everywhere, in a harmonious and seemless manner. The question mirrors that in a most elegant way.

The objective quality of life comprises all nonsubjective aspects of life related to external status and achievement, measured in terms of the norms that are dominant in the culture of the respondent. As theories of objective quality of life typically tend to be theories of lists, in which many things and qualities a person ought to possess are enumerated, we also constructed such a list. It is possible to make many other lists. However, as stated in our methodological criteria, these lists must be based on the theory or overall philosophy of life.

Based on the theory of realizing life potential, the list used in the SEQOL questionnaire was organized based on the division of life into domains, yet it is nothing more than a statement of how we see the norms of our Western societies.

## TWO HYBRID QUALITY-OF-LIFE RATING SCALES

We had to omit two rating scales (No. 4 on meaning in life and No. 5 on biological order) from the list of quality-of-life rating scales. These two questions related to something very deep in people that made it very difficult to express the response verbally. We felt this to be a deep loss, as our objective was to examine the truly essential things in life.

Without these two quality-of-life rating scales, the subjective and the objective parts of the spectrum of our integrative theory were not linked entirely well. We therefore tried to connect the two sides with two hybrid quality-of-life rating scales that combine the subjective and the objective so that they bridge the gap between these two aspects of the individual.

Both scales considered a number of objective aspects and asked the respondent to give his or her personal views on them. The first of these scales looked at life as divided into periods of time (time spent on family, work, and leisure hours, respectively). This corresponded to the concept of well being as it was developed and used in the 1960s. The concept does not play the same role today and has to some extent been replaced by the broader notion of the quality of life.

The other scale viewed the spatial division of life into various relations with one's self and the surrounding world. We defined five such relationships in which we may find ourselves: with one's self, with one's partner, with parents and children, with friends and acquaintances, and with society and nature. This scale used satisfaction as its subjective dimension, and was therefore called satisfaction with relationships.

The questions for both scales asked for the respondent's degree of satisfaction with these relationships. We had assumed that such questions as how satisfied people are would get closer to the core when asked in relation to something specific and tangible, and more superficial when people are asked to evaluate their lives at a general level as in questions 1 and 2 above.

The replacement of the two deep quality-of-life scales with evaluations of a number of objective aspects in life was far from ideal (as it was bound to be superficial). However, linguistic difficulties in reaching the most fundamental and deepest levels of the human being forced us to do this. Nevertheless, our tools and methods will probably improve in the future (possibly as a consequence of a more widespread use of such concepts as the quality of life and meaning in life, which would make more direct and searching questions possible), such that the ad hoc solution presented here will only be temporary. Still, the scores in *The Quality of Life in Denmark*[10] showed that the results were fairly consistent, and although the new hybrid instruments lacked depth, they did have the advantage of being easily accessible.

Yet another modification was necessary before the spectrum of the eight theories could be translated into rating scales that aptly illustrated the quality of life. This modification concerned the sequence of the graphic presentation.

The method chosen to present the results required a certain correlation between the scores of the various rating scales. The analysis of the initial results of the Quality of Life Survey showed that theory No. 7, “fulfillment of needs”, as an operationalized rating scale, was closer to the subjective than the objective end of the spectrum. In the statistical tables[10], fulfillment of needs was therefore placed as rating scale No. 4, after happiness, as fulfillment of needs was judged by the respondent by means of: "How well is need X fulfilled?"; that is, the question tended to remind the respondent of the subjective concept of feeling good.

The two hybrid rating scales are found in the SEQOL questionnaire and were thus placed as No. 5 and No. 6 in the analysis. Therefore, theory No. 6, “realizing life potential”, became rating scale No. 7 (the overall division into subjective, existential, and objective rating scales was, however, still maintained).

### THE INSTRUMENTS ARE CONSTANTLY UPDATED

The instruments and theories on which the Quality of Life Survey was based are constantly being updated. It is an ongoing process as culture, language, and consciousness of man are constantly developing. During the last decade, every Dane came to know the concept of global quality of life. We expect that the same will happen to the concept of purpose of life during the next decade. We continue to aim towards greater simplicity, precision, and depth in the questions, so that they are better able to explore the depths of human existence. We have not yet found a final form that enables us to fully rate the quality of life in practice, but it seems that the development of man and society in the western world will make it a lot easier to measure global quality of life according to the IQOL theory in the future. We hope that the several hundred questions we found necessary to adequately implement the theories of the Quality of Life Survey ultimately can be replaced by far fewer: ideally, these eight. These eight questions have not yet been evaluated. Therefore, they should not form the basis of a survey. However, the perspective is clear. If eight simple questions can accurately rate the quality of life as well as its depth, we have found an instrument of immense practical scope.

### ACKNOWLEDGMENTS

This study was supported by grants from The 1991 Pharmacy Foundation, as well as by supplementary grants from Goodwill-fonden, the JL-Foundation, E. Danielsen and Wife's

Foundation, Emmerick Meyer's Trust, the Frimodt-Heineken Foundation, the Hede Nielsen Family Foundation, Petrus Andersens Fond, Wholesaler C.P. Frederiksens Study Trust, Else & Mogens Wedell-Wedellsborg's Foundation, and IMK Almene Fond. We gratefully acknowledge the critical scrutiny and expert linguistic assistance of Ib Ravn, Ph.D. The research was approved by the Copenhagen Scientific Ethical Committee under number (KF)V.100.2123/91.

### BIOSKETCHES

**Søren Ventegodt, MD,** is the Director of the Quality of Life Research Center in Copenhagen, Denmark. He is also responsible for a Research Clinic for Holistic Medicine in Copenhagen and is a popular speaker throughout Scandinavia. He has published numerous scientific or popular articles and a number of books on holistic medicine, quality of life, and quality of working life. His most important scientific contributions are the comprehensive SEQOL questionnaire, the very short QOL5 questionnaire, the integrated quality-of-life (IQOL) theory, the holistic process theory, the life mission theory, and the Danish Quality of Life Research Survey, 1991–94 in cooperation with the University Hospital of Copenhagen and the late pediatric professor Bengt Zachau-Christiansen. E-mail: ventegodt@livskvalitet.org. Website: www.livskvalitet.org/

**Joav Merrick, MD, DMSc,** is Professor of Child Health and Human Development affiliated with the Zusman Child Development Center and Division of Community Health at the Ben Gurion University, Beer-Sheva, Israel and presently the Medical Director of the Division for Mental Retardation, Ministry of Social Affairs, Jerusalem and the Director of the National Institute of Child Health and Human Development. He has numerous publications in the field of child and human development, rehabilitation, intellectual disability, disability, health, welfare, abuse, advocacy and prevention. Dr. Merrick received the Peter Sabroe Child Award for outstanding work on behalf of Danish Children in 1985 and the International LEGO-Prize ("The Children's Nobel Prize") for an extraordinary contribution towards improvement in child welfare and well being in 1987. E-mail: jmerrick@internet-zahav.net. Website: www.nichd-israel.com

**Niels Jørgen Andersen, MSc,** Professor, Department of Innovation and Economic Organization, Norwegian School of Management. This department conducts research and provides teaching in central topics related to innovation, business development, management of global companies, business history, and economic organization. Research activities within the Department are related to four core subjects within the discipline: business history, cooperative organizations, business development and entrepreneurship, and finally studies of industries with a special focus on the electricity industry. He is also the dynamic chairman of the nonprofit organization Stiftelsen Holistisk Medisin Scandinavia, which aims to support the scientific development, research, and documentation of complementary and holistic medicine in Scandinavia. E-mail: niels.j.andersen@bi.no. Website: www.bi.no/users/fgl93013/

## APPENDIX

The SEQOL (Self-Evaluation of Quality of Life) Questionnaire

### About the Questionnaire

The concept of the quality of life has become a central concept in health care with many Doctors becoming more interested in increasing the quality of life of their patients. We have developed this questionnaire to examine the connection between quality of life and illness.

An (m) or an (f) after a question denotes that it is to be answered by men or women respectively.

At the end of the questionnaire we would like to know how you felt about filling in the questionnaire. Space has been provided for any comments you may have on the questionnaire or any personal reflections you may have on quality of life. All comments will be read carefully.

Please consider every question carefully before answering. It is important to the investigation that you answer honestly. We recommend that you fill out the questionnaire alone.

Please try to answer all the questions unless, of course, you find them too unpleasant, in which case you do not have to answer.

The answers to the questionnaire are anonymous and the information provided by these questionnaires is processed electronically and governed by the Danish Act on Information Held in Record Systems; this investigation is regulated by the Danish Data Surveillance Authority

### Instructions for filling out the Questionnaire

Please note that some of the questions deal with your subjective opinion whereas others deal with factual, objective matters. Most questions can be answered by simply circling the number that is placed next to the answer you have chosen (vertically or horizontally), for instance:

| | | |
|---|---|---|
| 9 **Sex:** | (1) | Female |
| | 2 | Male |

Where lines have been left open, please answer in words or numbers or give your opinion.

**The answers are anonymous**
**Please answer all the questions**
**Thank you for your help**

© 2003 The Quality of Life Research Centre. The questionnaire is hereby released for non-commercial, scientific use. Please apply for written permission for all commercial or non-scientific use to the Quality of Life Research Centre (www.livskvalitet.org).

## Personal information

Please note that you are **not** to write your name on the questionnaire! Your reply is anonymous.

**6-8 Today's date:**

**9 Sex:** 1 female
2 male

**10 Age:**____________years

**11 Height:**__________cm

**12 Weight**:__________kg

**13** What is your marital status?

| | | | |
|---|---|---|---|
| 1 | single | 4 | separated |
| 2 | living with partner | 5 | divorced |
| 3 | married | 6 | widowed |

**14** With **whom** do you live? (Please circle all that apply)

1 living alone
2 spouse or partner
3 own children
4 partner's children
5 adopted children
6 friends
7 biological parents
8 others
9 dog
10 cat
11 other domestic pet

**15-16** Where do you live?

| | | | |
|---|---|---|---|
| 1 | Copenhagen | 3 | In a village |
| 2 | In a large town (or suburb) of Copenhagen | 4 | In the country |

Geographical location:
**Postal Code**

**17** Type of housing:

1 own house
2 owner-occupied flat
3 cooperative
4 rented house
5 rented flat
6 rented room
7 student hostel (dormitory)
8 collective
9 others

**18** If you are currently receiving training or **education**, please specify the type:
_________________________________

**19-24** List any higher **education** or vocational **training** you have completed also indicating the duration of each course embarked upon (for example, vocational training - 30 months; apprenticeship - 30 months; university degree - 36 months):

____________________ months :____
____________________ months :____
____________________ months :____

**25-30** If you have a partner, please list the education and training that he or she has completed.

_____________________ months :____
_____________________ months :____
_____________________ months :____

**31-34** Your **occupation** . Please describe your occupation as precisely as possible (for example, farm owner instead of farmer, apprentice electrician instead of electrician):
_________________________________

What does your work consist of?_____________
________________________________________
_______

Please describe your partner's **occupation** (if you have a partner):
_________________________________

What does your partner’s work consist of?_____
__________________________

**35-36** What is your (and your partner’s) **occupational status**? (Please circle one number in each column. If you are unemployed please refer to the occupation you have had for the longest period of time.)

| | You | Your partner |
|---|---|---|
| self-employed | 1 | 1 |
| assistant in spouse's business | 2 | 2 |
| unskilled/ semi-skilled worker | 3 | 3 |
| skilled worker | 4 | 4 |
| homemaker | 5 | 5 |
| salaried employee or civil servant | 6 | 6 |
| student | 7 | 7 |
| pensioner | 8 | 8 |
| receiving welfare benefits | 9 | 9 |

**37-38** If you or your partner have **employees or subordinate**s, please state how many:
______yourself ______your partner

**39** Current sector in which you are **employed:**

| | | | |
|---|---|---|---|
| 1 | public | 3 | unemployed |
| 2 | private unemployed | 4 | other |

**40-41** What was the total income before taxes for yourself and your partner last year? (Please circle a number in each column)

| | yourself | your partner |
|---|---|---|
| 0 - 5.000 Euro/USD | 1 | 1 |
| 5.001 -10.000 Euro/USD | 2 | 2 |
| 10.001 -20.000 Euro/USD | 3 | 3 |
| 20.001 -40.000 Euro/USD | 4 | 4 |
| 40.001 -80.000 Euro/USD | 5 | 5 |
| over 80.000 Euro/USD | 6 | 6 |

**42** Does your **household** have any of the following durable consumer goods?

1 telephone
2 television
3 computer
4 videocassette recorder
5 washing machine
6 summer cottage
7 car
8 motorcycle
9 boat

**43** How would you describe your **current financial and material situation**?

1 very good
2 good
3 neither good nor poor
4 poor
5 very poor

## Lifestyle

**44** How many hours of exhausting **exercise** (heavy breathing and sweating) do you get **a week**? (including during work)

1 0 hours
2 about 30 minutes
3 about 1 hour
4 about 2 hours
5 3-7 hours
6 8 hours or more

**45** Your **social network**: how many close friends or family members do you have **daily contact** with (for example, see them or talk to them on the telephone)?

1 none
2 1 a day
3 2 a day
4 3 a day
5 4 or more

**46** Do you have a friend with whom you can discuss any subject - and do?

1 yes 2 not sure 3 no

**47** Your **eating habits**?
(Please circle the lines that apply to you)
1 I eat what I like
2 I think my diet is healthy
3 I think my diet is not healthy enough
4 I have a low-calorie diet to maintain or lose weight
5 I eat ready-to-eat dishes or fast food at least once a week
6 I am a vegetarian
7 Information campaigns influence my eating habits

**48-52** How much **alcohol** did you drink in the last complete weekday (Monday, Tuesday, Wednesday or Thursday)?

ordinary beer (number of 33-cl bottles): _______
strong beer (number of 33-cl bottles): _______
red or white wine (number of glasses): _______
dessert wine (number of glasses): _______
spirits (number of drinks): _______

**53-57** How much **alcohol** did you drink **in total last weekend** (including Friday, Saturday and Sunday)?

ordinary beer (number of 33-cl bottles): _______
strong beer (number of 33-cl bottles): _______
red or white wine (number of glasses): _______
dessert wine (number of glasses): _______
spirits (number of drinks): _______

**58** Was your **alcohol** consumption **typical** of your normal level in the past week?

1 yes
2 no, more than typical
3 no, less than typical

**59-63 Do you smoke**?

1 yes, daily
2 yes, now and then
3 no, I stopped less than a year ago
4 no, I stopped more than a year ago
5 no, I have never smoked

***If yes***, how much do you smoke on average:
number of cigarettes daily _____
number of cheroots daily _____
number of cigars daily _____
amount of smoking tobacco (grams) ____

**64** Have you ever tried any of the following **drugs**:

1 hash
2 LSD
3 psilosybin (mushrooms)
4 mescaline (cactus)
5 amphetamine, speed
6 cocaine
7 crack
8 ecstasy
9 methadone
10 heroin, morphine
11 tranquilizers with alcohol
12 none

## Illness

**65 -66** In relation to your **health**, how do you feel now? (Please circle a number in each column)

| | physical health | mental health |
|---|---|---|
| very well | 1 | 1 |
| well | 2 | 2 |
| neither well nor ill | 3 | 3 |
| ill | 4 | 4 |
| very ill | 5 | 5 |

**67** In the **last year**, how many days of work have you missed due to **illness**? ( Or, if you are unemployed, how many days would you have been too ill to work had you been in employment?)

| | | | |
|---|---|---|---|
| 1 | 0 - 3 days | 4 | 31 - 100 days |
| 2 | 4 - 10 days | 5 | 101 - 300 days |
| 3 | 11 - 30 days | 6 | 301 - 365 days |

**68.** If you suffer from illness or a health problem do you try to treat it by changing your outlook on life or your lifestyle?

1 no 2 not sure 3 yes

**69** Do you receive alternative health care now?

1 yes
2 no, but i did previously
3 no, and i have never received it

**70** Have you ever undergone an operation under full **anesthesia**?

1 no 2 not sure 3 yes

**71-86** Do you have any of the following health problems **now**? (Please circle a number in each row)

| | no | yes, somewhat | yes, a lot |
|---|---|---|---|
| pain or discomfort in shoulder or neck? | 1 | 2 | 3 |
| pain or discomfort in back or buttocks? | 1 | 2 | 3 |
| pain or discomfort in arms or hands, legs, knees, hips or joints? | 1 | 2 | 3 |
| headache | 1 | 2 | 3 |
| a rapid heart beat? | 1 | 2 | 3 |
| uneasiness nervousness, restlessness or anxiety? | 1 | 2 | 3 |
| stress | 1 | 2 | 3 |
| difficulty sleeping (insomnia)? | 1 | 2 | 3 |
| melancholy, depression or unhappiness? | 1 | 2 | 3 |
| tiredness? | 1 | 2 | 3 |
| stomach pain or stomachache? | 1 | 2 | 3 |
| indigestion, diarrhea or constipation? | 1 | 2 | 3 |
| eczema, rash, or itching? | 1 | 2 | 3 |
| cold, head cold, or cough? | 1 | 2 | 3 |
| difficulty in breathing or breathlessness? | 1 | 2 | 3 |
| discomfort in the sexual organs(f) | 1 | 2 | 3 |

**87** Did the health problems have anything to do with a certain illness or the treatment of it?

1 no 2 not sure 3 yes

**88-90** Do you have an **illness or disorder** for which you are receiving medical treatment now?

1 no 2 yes

***If yes:*** please state, as accurately as possible, the **diagnosis** of the illness or the nature of the handicap: (such as pollen allergy or depression )

__________________________________

What treatment or **medication** are you receiving? (Please write the brand name)

__________________________________

**91-123** Do you have any of the following illnesses or disorders **now**? (Please circle a number in each line)

| | no | yes |
|---|---|---|
| impaired hearing | 1 | 2 |
| impaired sight | 1 | 2 |
| back problems | 1 | 2 |
| diabetes | 1 | 2 |
| mental illnesses | 1 | 2 |
| epilepsy | 1 | 2 |
| paralysis in any part of the body | 1 | 2 |
| high blood pressure | 1 | 2 |
| coronary thrombosis | 1 | 2 |
| cerebral hemorrhage or cerebral thrombosis | 1 | 2 |
| chronic bronchitis | 1 | 2 |
| allergy (not hayfever) | 1 | 2 |
| hayfever | 1 | 2 |
| eczema | 1 | 2 |
| psoriasis | 1 | 2 |
| lupus erythematosus | 1 | 2 |
| ulcer | 1 | 2 |
| gallstones | 1 | 2 |
| kidney stones | 1 | 2 |
| HIV-positive | 1 | 2 |
| AIDS | 1 | 2 |
| cancer | 1 | 2 |
| congenital illnesses | 1 | 2 |
| amputation of arms or legs | 1 | 2 |
| venereal diseases | 1 | 2 |
| anorexia/bulimia | 1 | 2 |
| removal of a tumor in the breast (f) | 1 | 2 |
| removal of the entire breast (f) | 1 | 2 |
| menstrual problems (f) | 1 | 2 |
| diseases of the female sexual organs (not venereal diseases) (f) | 1 | 2 |
| had a cone biopsy performed (f) | 1 | 2 |
| had your uterus removed (f) | 1 | 2 |

**124** Have you ever had an abortion? (f)

1 no 3 yes, twice
2 yes, once 4 yes, 3 or more times

**125** Have you ever had a miscarriage? (f)

1 no 2 not sure 3 yes

## Sexuality

**126** Are you sexually active?

1 yes 2 no

**127** How **satisfied** are you with your sex life now?
1 very satisfied
2 satisfied
3 neither satisfied nor dissatisfied
4 dissatisfied
5 very dissatisfied

**128** Sexual orientation:

1 heterosexual (attracted to the opposite sex)
2 bisexual (attracted to both sexes)
3 homosexual (attracted to your own sex)

**129-137** Do you have sexual problems?

1 no
2 yes, but they are not connected to any prolonged illness or disability
3 yes, and they are connected to a prolonged illness or disability

***If yes,*** what is your problem: (please circle a number in each line )

| | yes | not sure | no |
|---|---|---|---|
| lack of a suitable sexual partner | 1 | 2 | 3 |
| reduced sexual desire | 1 | 2 | 3 |
| pain or discomfort during intercourse | 1 | 2 | 3 |
| unable to achieve orgasm | 1 | 2 | 3 |
| decreased ability to achieve erection(m) | 1 | 2 | 3 |
| premature ejaculation (m) | 1 | 2 | 3 |
| involuntary vaginal spasms severe enough to prevent intercourse (vaginism) (f) | 1 | 2 | 3 |
| other | 1 | 2 | 3 |

## Your Perception of Yourself

**138-173** Do you feel you are (please circle a number in each line):

| | yes | not sure | no |
|---|---|---|---|
| healthy | 1 | 2 | 3 |
| physically strong | 1 | 2 | 3 |
| in harmony with yourself | 1 | 2 | 3 |
| honest | 1 | 2 | 3 |
| sensitive | 1 | 2 | 3 |
| loving | 1 | 2 | 3 |
| beautiful | 1 | 2 | 3 |
| sexually attractive | 1 | 2 | 3 |
| loved | 1 | 2 | 3 |
| cheerful | 1 | 2 | 3 |
| open | 1 | 2 | 3 |
| sociable | 1 | 2 | 3 |
| good | 1 | 2 | 3 |
| free | 1 | 2 | 3 |
| ethical | 1 | 2 | 3 |
| with a sense of humor | 1 | 2 | 3 |
| intelligent | 1 | 2 | 3 |
| sensible | 1 | 2 | 3 |
| patient | 1 | 2 | 3 |
| psychologically strong | 1 | 2 | 3 |
| independent | 1 | 2 | 3 |
| strong-willed | 1 | 2 | 3 |
| competent | 1 | 2 | 3 |
| ambitious | 1 | 2 | 3 |
| involved | 1 | 2 | 3 |
| creative | 1 | 2 | 3 |
| responsible | 1 | 2 | 3 |
| conscientious | 1 | 2 | 3 |
| filled with fighting spirit | 1 | 2 | 3 |
| brave | 1 | 2 | 3 |
| self-confident | 1 | 2 | 3 |
| a success | 1 | 2 | 3 |
| profound | 1 | 2 | 3 |
| intuitive | 1 | 2 | 3 |
| spiritual | 1 | 2 | 3 |
| sincere | 1 | 2 | 3 |

## Your Perception of Life

**174-194** (please circle a number in each line)

| | yes | not sure | no |
|---|---|---|---|
| do you feel comfortable in your body? | 1 | 2 | 3 |
| do you accept yourself as you are? | 1 | 2 | 3 |
| are you happy most of the time? | 1 | 2 | 3 |
| do you find your life boring? | 1 | 2 | 3 |
| do you have negative thoughts about your life several times a day ? | 1 | 2 | 3 |
| do you often worry? | 1 | 2 | 3 |
| do you often regret your actions? | 1 | 2 | 3 |
| do you often feel lonely? | 1 | 2 | 3 |
| do you sometimes think of committing suicide? | 1 | 2 | 3 |
| do you think that other people speak well of you? | 1 | 2 | 3 |
| do you easily adjust to new situations? | 1 | 2 | 3 |
| do you see problems as a challenge? | 1 | 2 | 3 |
| do you feel your life is a success? | 1 | 2 | 3 |
| is there accordance between your thought words and actions? | 1 | 2 | 3 |
| do you feel free to choose what your life is going to be like? | 1 | 2 | 3 |
| do you feel happy and look forward to starting a new day when waking up in the morning? | 1 | 2 | 3 |
| do you blame others for your unhappiness? | 1 | 2 | 3 |
| do you forgive others easily? | 1 | 2 | 3 |
| do you feel that fate has treated you badly? | 1 | 2 | 3 |
| do you feel that your life has meaning? | 1 | 2 | 3 |
| do you feel that you are part of a greater whole? | 1 | 2 | 3 |

## Quality of Life

**195-210 My life** mainly focuses on:
(please circle a number in each line)

| | yes | not sure | no |
|---|---|---|---|
| surviving | 1 | 2 | 3 |
| making money | 1 | 2 | 3 |
| my job | 1 | 2 | 3 |
| being creative | 1 | 2 | 3 |
| sex | 1 | 2 | 3 |
| feeling good | 1 | 2 | 3 |
| fulfilling my needs | 1 | 2 | 3 |
| my relationship with my partner | 1 | 2 | 3 |
| my children or having children | 1 | 2 | 3 |
| having good friends | 1 | 2 | 3 |
| living in harmony with myself | 1 | 2 | 3 |
| self-realization | 1 | 2 | 3 |
| my career | 1 | 2 | 3 |
| making the world a better place | 1 | 2 | 3 |
| serving God | 1 | 2 | 3 |

others:

**211** How are you **feeling** now?

1 very good
2 good
3 neither good nor poor
4 poor
5 very poor

**212** How **satisfied** are you with your life now?

1 very satisfied
2 satisfied
3 neither satisfied nor dissatisfied
4 dissatisfied
5 very dissatisfied

**213** How **happy** are you now?

1 very happy
2 happy
3 neither happy nor unhappy
4 unhappy
5 very unhappy

**214** How would you assess the **quality of your life** now? (base your answer on what you believe quality of life to be)

1 very high
2 high
3 neither high nor low
4 low
5 very low

## Section A

The following questions concern your **current satisfaction** with life. Please answer all questions even if you do not have parents, a partner nor children.

**215-224** At present how satisfied are you with: (Please note that you can be satisfied even with, for instance, poor health.)

| | very satisatisfied | satis-fied | neither /nor | dis satis´ | very dis' |
|---|---|---|---|---|---|
| your **health**? | 1 | 2 | 3 | 4 | 5 |
| your **material and financial situation**? | 1 | 2 | 3 | 4 | 5 |
| your situation with your **partner?** | 1 | 2 | 3 | 4 | 5 |
| your **parents** (please answer even if your parents are not alive) ? | 1 | 2 | 3 | 4 | 5 |
| your situation with regard to **children** (please answer even if you do not have children)? | 1 | 2 | 3 | 4 | 5 |
| your relationships with **friends** | 1 | 2 | 3 | 4 | 5 |
| your relationships with **acquaintances**? | 1 | 2 | 3 | 4 | 5 |
| the **community** around you? | 1 | 2 | 3 | 4 | 5 |
| **nature** in your local environment? | 1 | 2 | 3 | 4 | 5 |
| your job situation | 1 | 2 | 3 | 4 | 5 |

**225** How well did Section A (questions 215-224) enable you to express **your quality of life?**

1 very well
2 well
3 neither well nor poorly
4 poorly
5 very poorly

## Section B

**226-227** Do you have a **partner**?

1 yes
2 not sure
3 no, but I have had one.
4 no, and I have never had one

***If yes,*** how long have you been with your present partner?

1 0-1 year
2 1-2 years
3 2-3 years
4 3-5 years
5 5-10 years
6 10-25 years
7 more than 25 years

**228-248** Do you have **children**?
(If necessary, please circle more than one number)

1 yes, biological children
2 yes, my partner's children
3 yes, adopted children
4 no, but I or my partner is pregnant
5 no, I/we are involuntarily childless
6 no, I/we have chosen not to have children
7 no

***If yes,*** please state the **sex** and **age** of your children:

| | boy | girl | age |
|---|---|---|---|
| 1st child (oldest) | 1 | 2 | |
| 2nd child | 1 | 2 | |
| 3rd child | 1 | 2 | |
| 4th child | 1 | 2 | |
| 5th child | 1 | 2 | |

Did you have these children with your present partner?

| | yes | no |
|---|---|---|
| 1st child (oldest) | 1 | 2 |
| 2nd child | 1 | 2 |
| 3rd child | 1 | 2 |
| 4th child | 1 | 2 |
| 5th child | 1 | 2 |

Are you living with your children?

| | yes | no |
|---|---|---|
| 1st child (oldest) | 1 | 2 |
| 2nd child | 1 | 2 |
| 3rd child | 1 | 2 |
| 4th child | 1 | 2 |
| 5th child | 1 | 2 |

**249** Were you **adopted** as a child?

1 no    2 not sure    3 yes

**250** Are both your **parents** (or adoptive parents) alive?
1 yes
2 no, only one of them
3 no, none of them
4 do not know

**251-260** How many **brothers and sisters** (or half brothers and half sisters) do you have and how old are they:

| | brother | sister | half brother | half sister | age |
|---|---|---|---|---|---|
| 1st sibling (oldest) | 1 | 2 | 3 | 4 | |
| 2nd sibling | 1 | 2 | 3 | 4 | |
| 3rd sibling | 1 | 2 | 3 | 4 | |
| 4th sibling | 1 | 2 | 3 | 4 | |
| 5th sibling | 1 | 2 | 3 | 4 | |

**261** Are you a **twin**?

1 no    3 yes, fraternal
2 no, triplet    4 yes, identical

**262 -296 A good relationship consists of**:

- good and useful communication
- an emotional fellowship
- being equal, open and honest toward each other
- being able to talk about all important issues
- a relationship that is not characterized by unresolved conflicts, suppression and dominance or sexual problems.

**Compared with** this, how good is your current **relationship** with: (Circle the numbers which suit your life. Please leave out people you have never been in contact with or who are dead)

| | Very good | good | Neither /nor | Poor | Very Poor |
|---|---|---|---|---|---|
| your present partner | 1 | 2 | 3 | 4 | 5 |
| your last partner | 1 | 2 | 3 | 4 | 5 |
| your previous partners | 1 | 2 | 3 | 4 | 5 |
| 1st child (oldest) | 1 | 2 | 3 | 4 | 5 |
| 2nd child | 1 | 2 | 3 | 4 | 5 |
| 3rd child | 1 | 2 | 3 | 4 | 5 |
| 4th child | 1 | 2 | 3 | 4 | 5 |
| 5th child | 1 | 2 | 3 | 4 | 5 |
| biological father | 1 | 2 | 3 | 4 | 5 |
| biological mother | 1 | 2 | 3 | 4 | 5 |
| stepfather | 1 | 2 | 3 | 4 | 5 |
| stepmother | 1 | 2 | 3 | 4 | 5 |
| adoptive father | 1 | 2 | 3 | 4 | 5 |
| adoptive mother | 1 | 2 | 3 | 4 | 5 |
| maternal grandmother | 1 | 2 | 3 | 4 | 5 |
| maternal grandfather | 1 | 2 | 3 | 4 | 5 |
| paternal grandmother | 1 | 2 | 3 | 4 | 5 |
| paternal grandfather | 1 | 2 | 3 | 4 | 5 |
| 1st sibling (oldest)) | 1 | 2 | 3 | 4 | 5 |
| 2nd sibling | 1 | 2 | 3 | 4 | 5 |
| 3rd sibling | 1 | 2 | 3 | 4 | 5 |
| 4th sibling | 1 | 2 | 3 | 4 | 5 |
| 5th sibling | 1 | 2 | 3 | 4 | 5 |
| partner's father | 1 | 2 | 3 | 4 | 5 |
| partner's mother | 1 | 2 | 3 | 4 | 5 |
| partner's siblings | 1 | 2 | 3 | 4 | 5 |
| partner's friends | 1 | 2 | 3 | 4 | |
| your grandchildren | 1 | 2 | 3 | 4 | 5 |
| your other relatives | 1 | 2 | 3 | 4 | 5 |
| your friends | 1 | 2 | 3 | 4 | 5 |
| your fellow workers | 1 | 2 | 3 | 4 | 5 |
| your acquaintances | 1 | 2 | 3 | 4 | 5 |
| yourself | 1 | 2 | 3 | 4 | 5 |
| the community around you | 1 | 2 | 3 | 4 | 5 |
| nature around you | 1 | 2 | 3 | 4 | 5 |

**297** How well does Section B (questions 226-296) enable you to express **your quality of life?**

1 very well
2 well
3 neither well nor poorly
4 poorly
5 very poorly

## Section C

The following questions deal with family, work and leisure time.

**298** How do you feel when you are at home?

1 very good
2 good
3 neither good nor poor
4 poor
5 very poor

**299** How do you feel when you are at work (if you do not have a job, how do you feel in your everyday life)?

1 very good
2 good
3 neither good nor poor
4 poor
5 very poor

**300** How do you feel in your **leisure time?**

1 very good
2 good
3 neither good nor poor
4 poorly
5 very poor

**301** How well does Section C (questions 297-299) enable you to express **your quality of life?**

1 very well
2 well
3 neither well nor poorly
4 poorly
5 very poorly

**302-303** How good is your work environment? (If you do not have any work, please go to the next question)

| | socially | physically |
|---|---|---|
| very good | 1 | 1 |
| good | 2 | 2 |
| neither good nor poor | 3 | 3 |
| poor | 4 | 4 |
| very poor | 5 | 5 |

**304** Do you feel that your work is **meaningful**? (If you do not have any work, please assess your normal daily activities)

1. very meaningful
2 meaningful
3 neither meaningful nor meaningless
4 meaningless
5 very meaningless

## Section D

The following questions deal with how well your needs are fulfilled.

**305** How well are your **basic biological needs** being fulfilled now? (that is. your physical needs such as food, clothing, heating, housing, sleep, sex, safety and security .)

1 very well
2 well
3 neither well nor poorly
4 poorly
5 very poorly
6 I do not have these needs

**306** How well are your **social needs** being fulfilled now?

(that is, your needs for warm human contact, recognition, friendship and social acceptance).

1 very well
2 well
3 neither well nor poorly
4 poorly
5 very poorly
6 I do not have these needs

**307** How well is your **need to be useful** being fulfilled now (that is, your need to help other people and your contribution to making the world a better place)?

1 very well
2 well
3 neither well nor poorly
4 poorly
5 very poorly
6. I do not have this need

**308** How well is your need for an **exciting and varied life** being fulfilled now (that is, your need to have an exciting and varied life with various experiences and actions)?

1 very well
2 well
3 neither well nor poorly
4 poorly
5 very poorly
6 I do not have these needs

**309** How well is your **need to realize you life potential** being fulfilled now (that is, your need to develop insight and the ability to take action that will enable you to live in ever increasing harmony with yourself)?

1 very well
2 well
3 neither well nor poor
4 poorly
5 very poorly

6 I do not have these needs

**310** How well does section D (questions 303-309) enable you to express **your quality of life?**

1 very well
2 well
3 neither well nor poorly
4 poorly
5 very poorly

**311** How well are **your needs** being fulfilled now

1. very well
2. well
3 neither well nor poorly
4 poorly
5 very poorly

**312** How many of the following societal norms do you fulfill now? (Societal norms here means having a) a job; b) education c) nuclear family; and d) at least 2 friends in whom you can confide everything and do so.)

1 all four
2 three of four
3 two of four
4 one of four
5 none

**313** How good is **your peace of mind** (your inner equilibrium and state of health) now?

1 very good
2 good
3 neither good nor poor
4 poor
5 very poor

**314** How well are you realizing your **deepest desires** now?

1. very well
2. well
3 neither well nor poorly
4 poorly
5 very poorly

**315** How **meaningful** is your life now?

1 very meaningful
2 meaningful
3 neither meaningful nor meaningless
4 meaningless
5 very meaningless

## Evaluation and criticism of the questionnaire (1-315)

**316** How **easy** did you find it to fill out the questionnaire?

1 very easy
2 easy
3 neither easy nor difficult
4 difficult
5 very difficult

**317** Did you find it **strenuous** to fill out the questionnaire?

1 not strenuous
2 strenuous
3 very strenuous

**318** How was your mood when you filled out the questionnaire

1 better than usual
2 usual
3 worse than usual

**319** How **long** did it take you to fill out the questionnaire?

1 less than 30 minutes
2 30 minutes to 1 hour
3 1-2 hours
4 2-4 hours
5 more than 4 hours

**320-321** While going through the questionnaire, did you come across any questions you felt we should not have asked? (**unethical** questions)?

1 no 2 not sure 3 yes

**If yes or in doubt,** please explain the reason:(please note the number of the question)

**322** Would you like to comment on any of the answers you have given, or the questions you have been asked?
Would you like to remark on the investigation or express your own view on quality of life? If so, please feel free to write any comments, favorable or otherwise, below.

**323-324** Did the questionnaire cover all the dimensions that relate to **your** quality of life?

1 yes 2 not sure 3 no

***If you answered no or not sure***, please state which topics have not been included:
